# Cardiovascular Risk Factors in Premature Ovarian Insufficiency using Hormonal Therapy

**DOI:** 10.1055/s-0043-1770088

**Published:** 2023-07-21

**Authors:** Gabriela Pravatta Rezende, Thamyse Dassie, Daniela Angerame Yela Gomes, Cristina Laguna Benetti-Pinto

**Affiliations:** 1 Universidade Estadual de Campinas, Campinas, SP, Brazil

**Keywords:** premature ovarian insufficiency, cardiovascular disease, secondary amenorrhea, hormonal therapy, cardiometabolic risk factors, insuficiência ovariana prematura, doença cardiovascular, amenorreia secundária, terapia hormonal, fatores de risco cardiometabólicos

## Abstract

**Objective** Premature ovarian insufficiency (POI) is characterized by early hypoestrogenism. An increased risk of cardiovascular (CV) disease is a long-term consequence of POI. A challenge of hormone therapy (HT) is to reduce the CV risk.

**Methods** Cross-sectional study with lipid profile analysis (total cholesterol, LDL-C, HDL-C, VLDL-C and triglycerides), blood glucose levels and arterial blood pressure of women with POI using HT, compared with age and BMI-matched women with normal ovarian function (controls).

**Results** The mean age and BMI of 102 POI patients using HT and 102 controls were 37.2 ± 6.0 and 37.3 ± 5.9 years, respectively; 27.0 ± 5.2 and 27.1 ± 5.4 kg/m ^2^ . There wasn't difference between groups in arterial systolic and diastolic blood pressure, blood glucose levels, total cholesterol, LDL-C, VLDL-C and triglycerides. HDL-C levels were significantly higher in the POI group (56.3 ± 14.6 and 52 ± 13.9mg/dL; *p* = 0.03). Arterial hypertension was the most prevalent chronic disease (12% in the POI group, 19% in the control group, p = ns), followed by dyslipidemia (6 and 5%, in POI and control women).

**Conclusion** Women with POI using HT have blood pressure levels, lipid and glycemic profile and prevalence of hypertension and dyslipidemia similar to women of the same age and BMI with preserved gonadal function, in addition to better HDL levels.

## Resumo

**Objetivo** A insuficiência ovariana prematura (IOP) é caracterizada pelo hipoestrogenismo precoce. Risco aumentado de doença cardiovascular (CV) é uma consequência a longo prazo da IOP e um desafio da terapia hormonal (TH) é reduzir o risco CV.

**Métodos** Estudo transversal com análise do perfil lipídico (colesterol total, LDL-C, HDL-C, VLDL-C e triglicerídeos), glicemia e pressão arterial de mulheres com IOP em uso de TH, em comparação a mulheres com função ovariana normal (controles) pareadas por idade e IMC.

**Resultados** A média de idade e IMC de 102 pacientes com IOP em uso de TH e 102 controles foi de 37,2 ± 6,0 e 37,3 ± 5,9 anos, respectivamente; 27,0 ± 5,2 e 27,1 ± 5,4 kg/m ^2^ . Não houve diferença entre os grupos na pressão arterial sistólica e diastólica, glicemia, colesterol total, LDL-C, VLDL-C e triglicerídeos. Os níveis de HDL-C foram significativamente maiores no grupo IOP (56,3 ± 14,6 e 52 ± 13,9mg/dL; *p* = 0,03). A hipertensão arterial foi a doença crônica mais prevalente (12% no grupo POI, 19% no grupo controle, p = ns), seguida da dislipidemia (6 e 5%, no grupo POI e controle).

**Conclusão** Mulheres com IOP em uso de TH apresentam níveis pressóricos, perfil lipídico e glicêmico e prevalência de hipertensão e dislipidemia semelhantes às mulheres da mesma idade e IMC com função gonadal preservada, além de melhores níveis de HDL.

## Introduction

Premature ovarian insufficiency (POI) is an unusual but important cause of sex steroid deficiency and infertility in women under 40. It is characterized by high FSH levels (>25 UI/L) and irregular menstrual cycles or lack of menstrual bleeding. POI affects 1% of women before the age of 40. 1 2 3 4 Many health complications associated with POI are directly related to ovarian hormone deficiency, particularly estrogen deficiency. 1 Presenting symptoms and health complications related to hypoestrogenism include menopausal symptoms, decreased bone mineral density, infertility, mood disorders, cognitive decline, a higher risk of developing type 2 diabetes mellitus (T2DM) or prediabetes, cardiovascular (CV) disease, decreased sexual function and impaired quality of life. 5 6 7 8 There is also some evidence of reduced life expectancy. 9 10

Estrogen has several regulatory cardiometabolic functions that reduce oxidative stress, vasoconstriction, atherosclerosis and ischemia. Furthermore, it modifies the hepatic metabolism of lipoproteins, reducing LDL-cholesterol (LDL-C) and increasing HDL-cholesterol (HDL-C) levels. 11 12 Compared with age-matched women with normal ovarian function, women with POI have diminished endothelial function and early signs of atherosclerosis. 13 14 15 The increased risk of CV disease and stroke underscore the role of hypoestrogenic status. In addition, POI women had higher levels of blood glucose, insulin resistance, systemic arterial blood pressure, along with increased inflammatory factors. 2 16 17 18 19 20 These data show that POI can be an independent risk factor for ischemic heart disease, and CV disease is possibly the main reason for a shorter life expectancy. 8 15

Unless a strong contraindication exists, HT is recommended for women with POI until the natural age of menopause for protection against the negative effects of hypoestrogenism. 2 In women after natural menopause, the use of HT has been associated with reduced levels of LDL-C, triglycerides (TG) and insulin resistance, in addition to increased levels of HDL-C. 21 22 23 24 Due to the complexity of estrogen and progestogen receptor systems, the benefits should be weighed and may differ in younger and healthier women. In POI, it is believed that HT can restore endothelial function usually within 6 months of treatment. 25 26 Nevertheless, there is insufficient data confirming other effects of the long-term use of HT to reduce cardiovascular risk in this specific population.

The current study was conducted to assess clinical and metabolic cardiovascular risk profile in women using HT diagnosed with POI, compared with a population of age-matched and BMI-matched women with preserved ovarian function.

## Methods

A cross-sectional study was conducted at the Endocrinological Gynecology Outpatient Clinic of the Department of Obstetrics and Gynecology of the University of Campinas. A convenience sample size was used, including women diagnosed with POI who were managed during a 24-month period. POI was diagnosed in women aged 40 or younger, without any menstrual periods or who had irregular menstrual cycles and at least two FSH values measuring higher than 25mUI/L, taken at least 4 weeks apart. Only women with secondary amenorrhea, normal karyotype and no history of oncology treatment, chemotherapy or radiotherapy were included. Furthermore, women belonging to the POI group should have been adequately using hormone therapy for at least 6 months to be included. Each woman from the POI group was matched for age (±2 years) and BMI class (<20kg/m ^2^ ; 20 to 24.9kg/m ^2^ ; 25 to 29.9 kg/m ^2^ ; 30 to 34.9 kg/m ^2^ ; 35 to 40kgm 2 ; > 40kg/m ^2^ ) to women with preserved gonadal function (control group), and follow-up visits in the Family Planning Unit at the same institution. Women from the control group were required to have spontaneous and regular menstrual cycles (between 24 and 38 days), a negative history of hormone use or at least in the preceding 6 months and serum FSH levels within the normal range. 27

In both groups, clinical parameters such as age, medical history (chronic hypertension, diabetes mellitus, dyslipidemia diagnosis), obstetric history, weight (kg), height (meters), BMI (kg/m ^2^ ), systolic blood pressure (SBP, mmHg) and diastolic blood pressure (DBP, mmHg) were evaluated. Metabolic parameters were also studied by laboratory measurements, after a 12-hour fast, blood glucose levels (mg/ dL), total cholesterol (mg/ dL), HDL-C (mg/dL), LDL-C (mg/dL), VLDL-cholesterol (mg/dL), TG (ml/dL), TSH (mUI/L), free T4 (ng/dL). Women under treatment for previously diagnosed chronic disease were not excluded (chronic hypertension, dyslipidemia, hypothyroidism, diabetes).

The analyzed variables were considered altered when: SBP ≥ 140mmHg; DBP ≥ 90mmHg; fasting glucose ≥ 100mg/dL; total cholesterol ≥ 200mg/dL; LDL-C ≥ 130mg/dL; HDL-C < 45 mg/dL; triglycerides ≥150ml/dL; TSH ≥ 5mU/L.

In the POI group, only age was evaluated at the time of POI diagnosis (in years) and duration of POI (calculated as years from the last period until study entry).

The HT of choice was: conjugated estrogen (0.625 mcg or 1.25 mcg, associated with medroxyprogesterone acetate); estradiol (1mg or 2mg, associated with norethisterone); combined oral contraceptive (30mcg of ethinylestradiol associated with levonorgestrel), and tibolone (2.5mg).

Categorical variables were described as the absolute frequency (n) and percentage (%), and numerical variables were described as mean and standard deviation values. To compare categorical variables between groups (POI and control), the Chi-square or Fisher's exact tests were used. The *t* -test or Mann-Whitney test was used to compare numerical variables. To compare categorical variables, a paired analysis was performed using the McNemar test. A paired *t* -test was used to compare quantitative variables. A 5% significance level was adopted. The computer program used was SAS (Statistical Analysis System), version 9.4 for Windows.

This study was approved by the Ethics Review Board of the institution (CAAE n° 08623412.7.0000.5404).

## Results

**Fig. 1:**
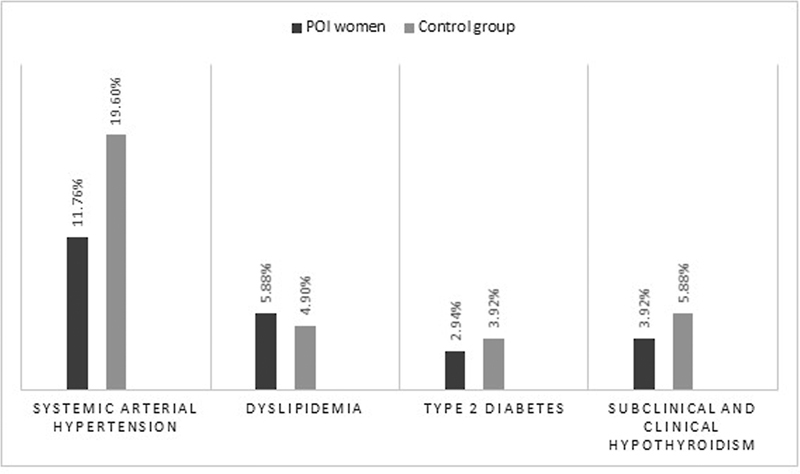
Prevalence of Chronic Diseases in women with POI and control group.

**Fig. 2:**
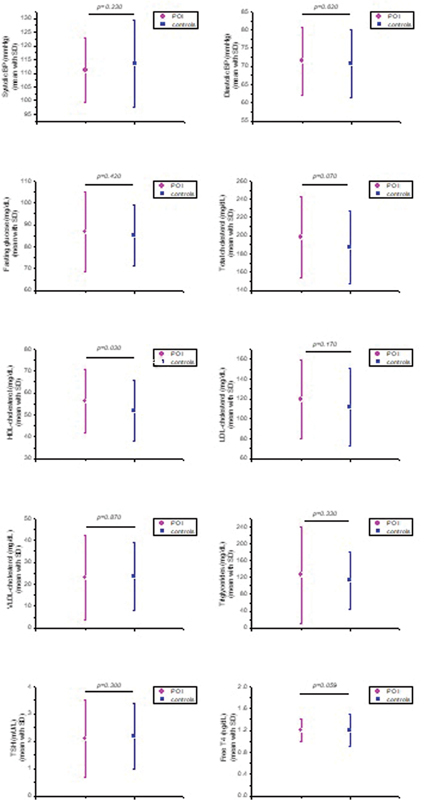
Comparative analysis of clinical and laboratory parameters between women with POI ( *N* = 102) and those with preserved gonadal function ( *n* = 102). * Student 's *t* -test.

A total of 204 women were included in the study. Of these women, 102 had POI and were taking HT and 102 women belonged to the control group (with preserved gonadal function). The mean age and mean BMI, for POI and controls, were 37.2 ± 6.0 and 37.3 ± 5.9 years, respectively; 27.0 ± 5.2 kg/m ^2^ and 27.1 ± 5.4 kg/m ^2^ , with no difference between groups. Ovarian insufficiency was established at age 31.9 ± 7.8 years on average and the time between diagnosis and study inclusion was 5.3 ± 5.6 years. HT containing conjugated estrogen was used by 63 women, estradiol by 33 women, combined oral contraceptives by 3 women, as well tibolone (3 women). Women with POI conceived less often and gave fewer births than women in the control group, and there was no difference in the number of miscarriages ( Table 1 ). The groups did not differ in mean levels of SBP (111.1 ± 11.7 of the POI group; 113.5 ± 16 mm Hg of the control group), DBP (71.5 ± 9.3 and 70.8 ± 9.4 mm Hg), blood glucose levels (86.8 ± 18.4 and 85.2 ± 14.1 mg/dL), total cholesterol (198.1 ± 44.6 and 187.3 ± 39.7 mg/dL), HDL-C (56.3 ± 14.6 and 52.0 ± 13.9 mg/dL), LDL-C (119.3 ± 39.6 and 111.8 ± 38.8 mg/dL), VLDL-C (23.0 ± 19.1 and 23.5 ± 15.7 mg/dL), triglycerides (126 ± 115 and 112.9 ± 67.7 mg/dL) and thyroid function (TSH 2.1 ± 1.4 and 2.2 ± 1.2 mU/L, respectively; p= NS; Table 2 ). There were also no differences between the mean metabolic parameters evaluated, fasting blood glucose, total cholesterol, LDL-C and triglycerides. Serum HDL-C levels were significantly higher in the POI group (56.3 ± 14.6 and 52 ± 13.9; *p* = 0.03). Thyroid function parameters were similar in both groups ( Fig. 1 and 2 ).

**Table 1:** Characteristics of premature ovarian insufficiency (POI) and women with preserved gonadal function (control group)

	POI Group ( *n* = 102)	Control Group( *n* = 102)	p-value (*)
Age (Years)	37.2 ± 6.0	37.3 ± 5.9	0.210
Weight (kg)	66.2 ± 15.1	70.0 ± 14.7	0.002
Height (m)	1.5 ± 0.2	1.6 ± 0.1	0.010
BMI (kg/m2)	27.0 ± 5.2	27.1 ± 5.4	0.490
Pregnancies	1.7 ± 1.6	2.3 ± 1.7	0.007
Deliveries	1.4 ± 1.3	1.9 ± 1.3	0.004
Miscarriage	0.3 ± 0.9	0.3 ± 0.9	0.880
FSH (mUI/mL)	85.3 ± 38.2	6.7 ± 8.9	<0.0001

Abbreviations: BMI, Body Mass Index; FSH, Follicle-stimulating hormone.

*Student *t* -test.

**Table 2:** Clinical parameters of women with POI and control group

	POI Group ( *n* = 102)	Control Group ( *n* = 102)	p-value
Systolic blood pressure levels (mmHg)	111.1 ± 11.7	113.5 ± 16	ns
Total cholesterol (mg/dL)	198.1 ± 44.6	187.3 ± 39.7	ns
HDL-cholesterol (mg/dL)	56.3 ± 14.6	52.0 ± 13.9	ns
LDL-cholesterol (mg/dL)	119.3 ± 39.6	111.8 ± 38.8	ns
Thyroid function (mU/L)	2.1 ± 1.4	2.2 ± 1.2	ns

ns, not significant.

**Fig. 3:**
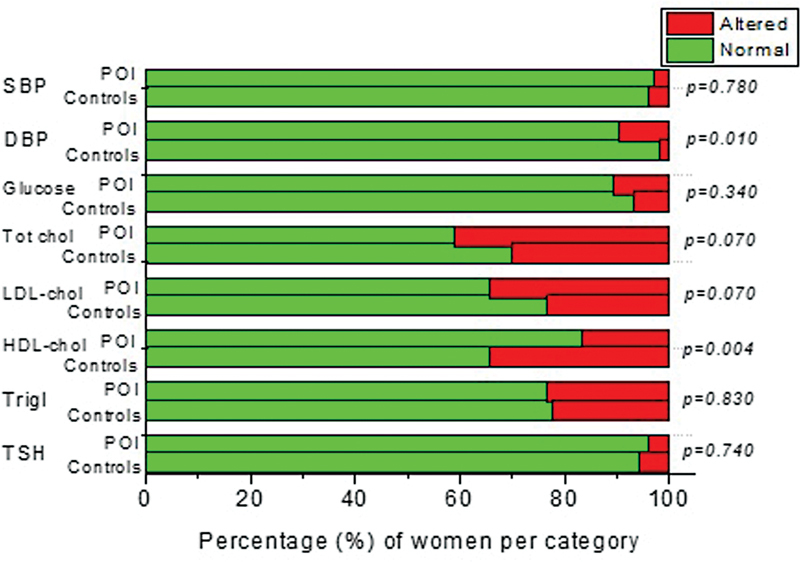
Parameters associated with the most prevalent chronic diseases (Chronic hypertension: *n* = 12 and 20 in the POI and control groups; Diabetes Mellitus: *n* = 3 and 4 in the POI and control group; Dyslipidemia: *n* = 6 and 5 in the POI and control group), classified as normal or altered. *Mann-Whitney test for a mean comparison between groups; POI (premature ovarian insufficiency).

Among chronic diseases, systemic arterial hypertension was the most prevalent condition in both groups (11.76% in the POI group; 19.6% in the control group), followed by dyslipidemia, diagnosed in 5.88% of women with POI and 4.90% of women with preserved gonadal function, without any statistical difference ( Fig. 1 ). T2DM was diagnosed in 2.94% of women with POI and in 3.92% of controls, while subclinical and clinical hypothyroidism was present in 3.92% of women with POI and 5.88% in the control group, without any difference between groups ( Fig. 2 ). Adequate disease control, assessed by measurements of blood pressure, blood glucose levels, cholesterol, triglycerides and TSH within the normal range, also showed no statistical difference between groups, with the exception of diastolic blood pressure, where levels above normal were more commonly found in women with POI ( Fig. 3 ).

## Discussion

This study evaluated parameters associated with increased cardiovascular risk in predominantly overweight women (mean age, 37 years) with POI taking hormone therapy, in relation to age-matched and BMI-matched women with preserved ovarian function. The groups were similar in the majority of evaluated parameters. However, serum HDL-C levels were significantly higher in the POI group. Chronic arterial hypertension and dyslipidemia were the most prevalent diseases in both groups. BMI averages close to 27kg/m ^2^ revealed a high prevalence of overweight and obese women, in agreement with values found among the female population in the world and in Brazil. 28

Cardiovascular disease is currently the main cause of morbidity and mortality among women. 29 POI is considered an independent albeit modest cardiovascular risk factor, especially for ischemic heart disease and overall CV disease, with the exclusion of stroke. 16 Epidemiological data suggest that life expectancy in women with POI is 2 years shorter than in women with natural menopause (at the usual age). Although many gaps remain in the current knowledge of the long-term consequences of hypoestrogenism for young women, as well as the control of the effects of estrogen replacement therapy, the available literature suggests that hormone therapy can reduce morbidity and mortality in this population. 30 31 There is evidence showing similar levels of glucose, insulin, HOMA-IR, LDL-C and TG among women with POI using HT, compared with controls with preserved ovarian function. 17 18 However, despite substantial evidence that treatment with estrogen improves vascular endothelial function in postmenopausal women, studies on the impact of HT on the cardiovascular system of women with POI are limited. 26 31

Since early hypoestrogenism seems to be associated with a higher mortality rate due to cardiac ischemia, this risk is known to increase in association with uncontrolled blood pressure levels. 10 23 29 Results have shown that women diagnosed with previous hypertension that experience menopause at a physiological age and initiate HT in the first 10 years of amenorrhea or before the age of 60 may have improved blood pressure levels. However, the effect of hormone replacement therapy on blood pressure control in women with POI is less widely known. 12 Some data show a similar effect, especially in those using formulations containing natural estrogen. 32 In our study, systemic arterial hypertension was the most prevalent chronic disease among all women evaluated (12 women in the POI group, 20 in the control group). There was no statistical difference between the two groups, and both groups were adequately controlled.

Dyslipidemia was the second most prevalent condition found in both groups studied. However, in women with POI, HDL-C levels were significantly higher, which is in agreement with the available literature on the subject. 17 20 Oral HT containing estrogen in postmenopausal women is associated with a reduction in total cholesterol and LDL-C and an increased HDL-C level, an effect that was not observed in transdermal hormone therapy. 24 25 26 Regarding POI, the data are scarce, but studies conducted with women undergoing surgical menopause and transdermal hormone replacement demonstrated an improvement in HDL-C levels, compared with those undergoing oophorectomy who did not use HT. 33 34 There is also evidence that endothelial function is established within six months of hormone replacement in women with POI. In addition to an improvement in lipid profile, a decrease in cardiovascular risk may also be associated. 15 22 34

There is controversy in the literature surrounding other metabolic parameters. Regarding TG levels, evidence suggests that women with POI who do not use HT more commonly have higher triglyceride levels than women with preserved gonadal function, while divergent results were obtained by other authors. 10 14 16 33 In women with natural menopause, oral HT can increase triglyceride levels. 25 Our results did not show any difference in lipid metabolism between the two groups, although the oral route was chosen for the administration of HT.

Glucose metabolism is also influenced by hypoestrogenism. There is evidence that a higher prevalence of insulin-dependent diabetes mellitus occurs among women with POI. In women who develop T2DM during the period of hypoestrogenism, HT is associated with a greater insulin sensitivity. 26 29 30 In the current study, the prevalence of T2DM was similar between both groups analyzed, just like fasting glucose levels.

Our results showed that women with POI who had been using HT did not present worse cardiometabolic markers or comorbidities, in comparison to age-matched and BMI-matched women with preserved ovarian function. These results point toward the same direction as the available literature, although most studies were conducted with very small sample sizes (less than 20 women). Thus, the strength of our study is the expressive sample included, especially considering that the prevalence of POI is low. A recent meta-analysis was performed with a total of 21 studies involving 1573 women with POI. Most of the included studies have around 30–80 subjects. However, most studies do not differentiate between women with POI using and not using HT. According to these authors, analysis between POI women using and not using hormone therapy was not available because most included studies didn't mention whether the women used hormone therapy, which reinforce that our results can collaborate to reduce the gap in the evidences. 35 All women were using HT and were matched by age and BMI to control group women, which are confounding factors that can influence the variables that were analyzed. Furthermore, women diagnosed with previous comorbidities that have a potential cardiovascular risk were not excluded to avoid an important selection bias. Limitations of the study were the characteristics of design (cross-sectional) that do not allow a cause-and-effect relationship, in addition to the lack of a control POI group that does not use HT. Nevertheless, there are ethical restrictions on with holding hormone treatment from these women, unless they refuse to take it.

## Conclusion

Women with POI using HT had levels of blood pressure, lipid profile and blood glucose, similar to those of age-matched and BMI-matched women with preserved gonadal function. Comorbidities such as systemic arterial hypertension, diabetes and thyroid dysfunction were similar between both groups. Overweight and obesity should be viewed as comorbid conditions. These risk factors were equally prevalent in both groups.
